# 2-(4-Meth­oxy­phen­oxy)pyrazine

**DOI:** 10.1107/S1600536810031946

**Published:** 2010-08-18

**Authors:** Shah Bakhtiar Nasir, Zanariah Abdullah, Azizah Mainal, Zainal A. Fairuz, Seik Weng Ng, Edward R. T. Tiekink

**Affiliations:** a Department of Chemistry, University of Malaya, 50603 Kuala Lumpur, Malaysia

## Abstract

In the title compound, C_11_H_10_N_2_O_2_, the aromatic rings are almost orthogonal to each other [dihedral angle = 86.97 (8)°], with the benzene ring orientated to face one of the pyrazine N atoms. In the crystal, centrosymmetrically related pairs are connected *via* pairs of C—H⋯π inter­actions and the dimeric units thus formed pack into undulating layers that stack along the *a* axis.

## Related literature

For background to the fluorescence properties of compounds related to the title compound, see: Kawai *et al.* (2001 ▶); Abdullah (2005 ▶). For a related structure, see: Nasir *et al.* (2010 ▶).
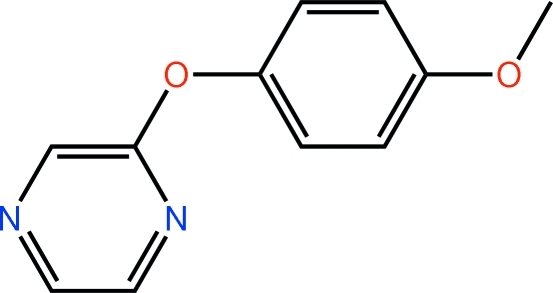


## Experimental

### 

#### Crystal data

C_11_H_10_N_2_O_2_
*M* *_r_* = 202.21
Monoclinic, 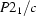

*a* = 5.8783 (2) Å
*b* = 10.9298 (4) Å
*c* = 15.6430 (6) Å
β = 97.109 (2)°
*V* = 997.32 (6) Å^3^
*Z* = 4
Mo *K*α radiation
μ = 0.10 mm^−1^
*T* = 293 K
0.35 × 0.20 × 0.10 mm

#### Data collection

Bruker SMART APEX CCD diffractometer
5515 measured reflections
1743 independent reflections
1244 reflections with *I* > 2σ(*I*)
*R*_int_ = 0.026

#### Refinement

*R*[*F* ^2^ > 2σ(*F* ^2^)] = 0.036
*wR*(*F* ^2^) = 0.100
*S* = 1.05
1743 reflections
138 parameters
H-atom parameters constrained
Δρ_max_ = 0.13 e Å^−3^
Δρ_min_ = −0.12 e Å^−3^

### 

Data collection: *APEX2* (Bruker, 2009 ▶); cell refinement: *SAINT* (Bruker, 2009 ▶); data reduction: *SAINT*; program(s) used to solve structure: *SHELXS97* (Sheldrick, 2008 ▶); program(s) used to refine structure: *SHELXL97* (Sheldrick, 2008 ▶); molecular graphics: *ORTEP-3* (Farrugia, 1997 ▶) and *DIAMOND* (Brandenburg, 2006 ▶); software used to prepare material for publication: *publCIF* (Westrip, 2010 ▶).

## Supplementary Material

Crystal structure: contains datablocks global, I. DOI: 10.1107/S1600536810031946/hb5602sup1.cif

Structure factors: contains datablocks I. DOI: 10.1107/S1600536810031946/hb5602Isup2.hkl

Additional supplementary materials:  crystallographic information; 3D view; checkCIF report

## Figures and Tables

**Table 1:** Hydrogen-bond geometry (Å, °) *Cg*1 is the centroid of the N1,N2,C1–C4 ring.

*D*—H⋯*A*	*D*—H	H⋯*A*	*D*⋯*A*	*D*—H⋯*A*
C9—H9⋯*Cg*1^i^	0.93	2.87	3.6326 (18)	140

Symmetry code: (i) 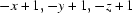.

**Fig. 1.**
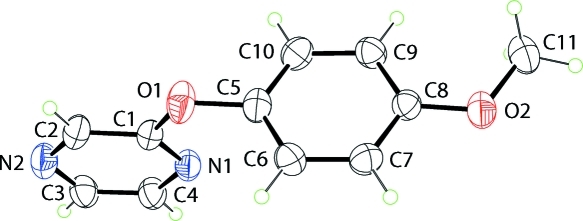
The molecular structure of (I) showing displacement ellipsoids at the 35% probability level.

**Fig. 2.**
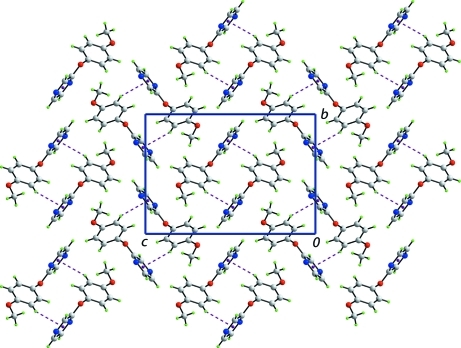
Undulating supramolecular layer in (I) showing C–H···π interactions (purple dashed lines) between centrosymmetrically related molecules.

**Fig. 3.**
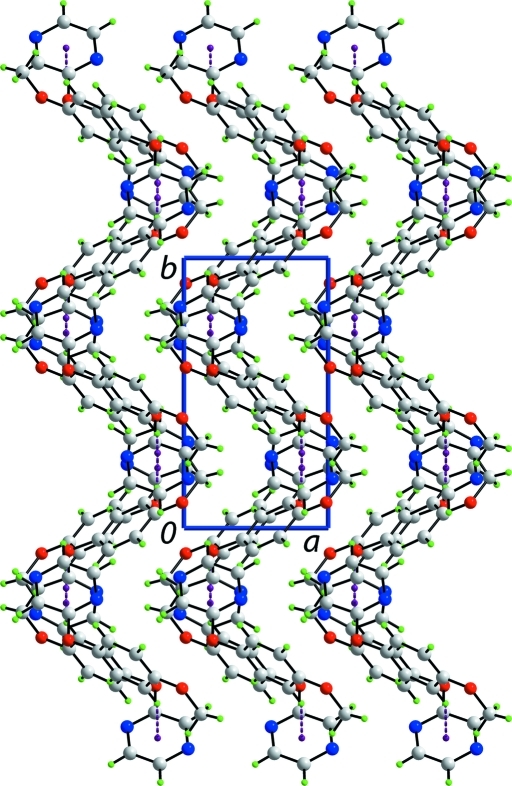
Unit-cell contents shown in projection down the c axis in (I), highlighting the stacking of undulating layers. The C–H···π interaction are shown as purple dashed lines.

## supplementary crystallographic information

### Comment

On-going structural studies of heterocyclic N-containing derivatives (Nasir *et al.*, 2010) are motivated by an investigation of their fluorescence properties (Kawai *et al.*, 2001; Abdullah, 2005). In this connection, the title pyrazine derivative, (I), was investigated.

With respect to the benzene ring, the pyrazine ring occupies an orthogonal position with the dihedral angle formed between the rings being 86.97 (8) °. The least-squares plane through the pyrazine ring is aligned along the C5···C8 axis of the benzene ring, an arrangement that allows the benzene ring to be directed towards the pyrazine-N1 atom. The C11–O2–C8–C7 torsion angle of -176.93 (14) ° indicates the methoxy group is co-planar with the benzene ring to which it is attached.

The most prominent intermolecular interaction operating in the crystal structure of (I) is of the type C–H···π. This occurs between centrosymmetrically related molecules and involves a benzene-H and the pyrazine ring, Table 1. The dimeric aggregates pack into undulating layers in the *bc* plane, Fig. 2, which stack along the *a* axis, Fig. 3.

### Experimental

2-Chloropyrazine (2.5 g, 45 mmol) and 4-methoxyphenol (5.6 g, 45 mmol) were refluxed in THF (10 ml) for 5 h. The residue was dissolved in minimum of water (10 ml) and extracted with ether (3 *x* 10 ml). The ethereal layer was washed with water and dried over anyhydrous sodium sulfate. Recrystallization from ethyl acetate yielded colourless blocks of (I) after a few days.

### Refinement

Carbon-bound H-atoms were placed in calculated positions (C—H 0.93 to 0.96 Å) and were included in the refinement in the riding model approximation, with *U*_iso_(H) set to 1.2 to 1.5*U*_equiv_(C). Some disorder was noted in the benzene ring (manifested in the shorter than usual average C–C bond distance of 1.37 Å). However, multiple sites were not resolved for this ring.

### Crystal data

**Table:**

C_11_H_10_N_2_O_2_	*F*(000) = 424
*M**_r_* = 202.21	*D*_x_ = 1.347 Mg m^−^^3^
Monoclinic, *P*2_1_/*c*	Mo *K*α radiation, λ = 0.71073 Å
Hall symbol: -P 2ybc	Cell parameters from 1293 reflections
*a* = 5.8783 (2) Å	θ = 2.3–22.4°
*b* = 10.9298 (4) Å	µ = 0.10 mm^−^^1^
*c* = 15.6430 (6) Å	*T* = 293 K
β = 97.109 (2)°	Block, colourless
*V* = 997.32 (6) Å^3^	0.35 × 0.20 × 0.10 mm
*Z* = 4	

### Data collection

**Table:**

Bruker SMART APEX CCD diffractometer	1244 reflections with *I* > 2σ(*I*)
Radiation source: fine-focus sealed tube	*R*_int_ = 0.026
graphite	θ_max_ = 25.0°, θ_min_ = 2.3°
ω scans	*h* = −6→6
5515 measured reflections	*k* = −12→12
1743 independent reflections	*l* = −18→18

### Refinement

**Table:**

Refinement on *F*^2^	Secondary atom site location: difference Fourier map
Least-squares matrix: full	Hydrogen site location: inferred from neighbouring sites
*R*[*F*^2^ > 2σ(*F*^2^)] = 0.036	H-atom parameters constrained
*wR*(*F*^2^) = 0.100	*w* = 1/[σ^2^(*F*_o_^2^) + (0.0486*P*)^2^ + 0.0504*P*] where *P* = (*F*_o_^2^ + 2*F*_c_^2^)/3
*S* = 1.05	(Δ/σ)_max_ = 0.001
1743 reflections	Δρ_max_ = 0.13 e Å^−^^3^
138 parameters	Δρ_min_ = −0.12 e Å^−^^3^
0 restraints	Extinction correction: *SHELXL97* (Sheldrick, 2008), Fc^*^=kFc[1+0.001xFc^2^λ^3^/sin(2θ)]^-1/4^
Primary atom site location: structure-invariant direct methods	Extinction coefficient: 0.042 (4)

### Special details

**Table:**

Geometry. All e.s.d.'s (except the e.s.d. in the dihedral angle between two l.s. planes) are estimated using the full covariance matrix. The cell e.s.d.'s are taken into account individually in the estimation of e.s.d.'s in distances, angles and torsion angles; correlations between e.s.d.'s in cell parameters are only used when they are defined by crystal symmetry. An approximate (isotropic) treatment of cell e.s.d.'s is used for estimating e.s.d.'s involving l.s. planes.
Refinement. Refinement of *F*^2^ against ALL reflections. The weighted *R*-factor *wR* and goodness of fit *S* are based on *F*^2^, conventional *R*-factors *R* are based on *F*, with *F* set to zero for negative *F*^2^. The threshold expression of *F*^2^ > σ(*F*^2^) is used only for calculating *R*-factors(gt) *etc*. and is not relevant to the choice of reflections for refinement. *R*-factors based on *F*^2^ are statistically about twice as large as those based on *F*, and *R*- factors based on ALL data will be even larger.

### Fractional atomic coordinates and isotropic or equivalent isotropic displacement parameters (Å^2^)

**Table:**

	*x*	*y*	*z*	*U*_iso_*/*U*_eq_	
O1	0.80138 (19)	0.41576 (11)	0.38163 (8)	0.0650 (4)	
O2	0.0132 (2)	0.59432 (11)	0.19506 (8)	0.0638 (4)	
N1	0.6059 (2)	0.26379 (12)	0.44535 (9)	0.0494 (4)	
N2	1.0286 (2)	0.18218 (14)	0.52555 (9)	0.0599 (4)	
C1	0.7989 (3)	0.31704 (15)	0.43404 (10)	0.0442 (4)	
C2	1.0109 (3)	0.27760 (17)	0.47408 (11)	0.0544 (5)	
H2	1.1426	0.3197	0.4642	0.065*	
C3	0.8320 (3)	0.12604 (17)	0.53716 (12)	0.0579 (5)	
H3	0.8364	0.0578	0.5729	0.069*	
C4	0.6268 (3)	0.16632 (15)	0.49808 (12)	0.0559 (5)	
H4	0.4948	0.1247	0.5082	0.067*	
C5	0.5920 (3)	0.45725 (15)	0.33748 (11)	0.0489 (4)	
C6	0.5262 (3)	0.41868 (15)	0.25481 (12)	0.0548 (5)	
H6	0.6122	0.3603	0.2299	0.066*	
C7	0.3328 (3)	0.46665 (16)	0.20906 (11)	0.0554 (5)	
H7	0.2881	0.4407	0.1529	0.066*	
C8	0.2035 (3)	0.55333 (14)	0.24574 (10)	0.0458 (4)	
C9	0.2718 (3)	0.59135 (15)	0.32910 (11)	0.0516 (5)	
H9	0.1863	0.6494	0.3547	0.062*	
C10	0.4674 (3)	0.54303 (16)	0.37434 (10)	0.0548 (5)	
H10	0.5145	0.5692	0.4303	0.066*	
C11	−0.1195 (3)	0.68815 (16)	0.22838 (12)	0.0637 (5)	
H11A	−0.2509	0.7059	0.1875	0.096*	
H11B	−0.0278	0.7606	0.2386	0.096*	
H11C	−0.1691	0.6611	0.2815	0.096*	

### Atomic displacement parameters (Å^2^)

**Table:**

	*U*^11^	*U*^22^	*U*^33^	*U*^12^	*U*^13^	*U*^23^
O1	0.0391 (7)	0.0747 (9)	0.0790 (9)	−0.0056 (6)	−0.0008 (6)	0.0292 (7)
O2	0.0585 (8)	0.0683 (8)	0.0606 (8)	0.0168 (6)	−0.0080 (6)	−0.0020 (6)
N1	0.0386 (8)	0.0495 (8)	0.0588 (9)	0.0018 (6)	0.0009 (6)	0.0061 (7)
N2	0.0492 (9)	0.0715 (10)	0.0566 (9)	0.0120 (8)	−0.0032 (7)	0.0042 (8)
C1	0.0373 (9)	0.0497 (10)	0.0447 (9)	0.0013 (7)	0.0010 (7)	0.0008 (8)
C2	0.0382 (10)	0.0698 (12)	0.0539 (10)	0.0005 (8)	0.0003 (8)	−0.0011 (10)
C3	0.0598 (12)	0.0538 (11)	0.0584 (11)	0.0097 (9)	0.0007 (9)	0.0081 (9)
C4	0.0482 (11)	0.0502 (10)	0.0682 (12)	0.0001 (8)	0.0035 (9)	0.0089 (9)
C5	0.0404 (10)	0.0500 (10)	0.0553 (10)	−0.0039 (8)	0.0028 (8)	0.0146 (9)
C6	0.0483 (11)	0.0525 (11)	0.0641 (11)	0.0066 (8)	0.0086 (9)	−0.0053 (9)
C7	0.0571 (12)	0.0596 (11)	0.0484 (10)	0.0020 (9)	0.0018 (8)	−0.0104 (9)
C8	0.0450 (10)	0.0440 (9)	0.0476 (9)	−0.0007 (7)	0.0026 (8)	0.0036 (8)
C9	0.0553 (11)	0.0511 (10)	0.0490 (10)	0.0072 (8)	0.0082 (8)	−0.0028 (8)
C10	0.0591 (11)	0.0640 (11)	0.0401 (9)	−0.0019 (9)	0.0017 (8)	−0.0008 (9)
C11	0.0519 (11)	0.0621 (11)	0.0772 (13)	0.0109 (9)	0.0088 (10)	0.0112 (10)

### Geometric parameters (Å, °)

**Table:**

O1—C1	1.3563 (19)	C5—C10	1.361 (2)
O1—C5	1.4096 (19)	C5—C6	1.370 (2)
O2—C8	1.3639 (18)	C6—C7	1.371 (2)
O2—C11	1.426 (2)	C6—H6	0.9300
N1—C1	1.3062 (19)	C7—C8	1.383 (2)
N1—C4	1.344 (2)	C7—H7	0.9300
N2—C2	1.314 (2)	C8—C9	1.380 (2)
N2—C3	1.341 (2)	C9—C10	1.378 (2)
C1—C2	1.392 (2)	C9—H9	0.9300
C2—H2	0.9300	C10—H10	0.9300
C3—C4	1.357 (2)	C11—H11A	0.9600
C3—H3	0.9300	C11—H11B	0.9600
C4—H4	0.9300	C11—H11C	0.9600
			
C1—O1—C5	118.56 (12)	C5—C6—H6	120.2
C8—O2—C11	118.05 (13)	C7—C6—H6	120.2
C1—N1—C4	114.95 (14)	C6—C7—C8	120.56 (16)
C2—N2—C3	116.22 (15)	C6—C7—H7	119.7
N1—C1—O1	120.72 (14)	C8—C7—H7	119.7
N1—C1—C2	123.03 (16)	O2—C8—C9	124.90 (15)
O1—C1—C2	116.26 (14)	O2—C8—C7	115.92 (15)
N2—C2—C1	121.37 (16)	C9—C8—C7	119.17 (16)
N2—C2—H2	119.3	C10—C9—C8	119.77 (15)
C1—C2—H2	119.3	C10—C9—H9	120.1
N2—C3—C4	121.60 (17)	C8—C9—H9	120.1
N2—C3—H3	119.2	C5—C10—C9	120.31 (16)
C4—C3—H3	119.2	C5—C10—H10	119.8
N1—C4—C3	122.83 (16)	C9—C10—H10	119.8
N1—C4—H4	118.6	O2—C11—H11A	109.5
C3—C4—H4	118.6	O2—C11—H11B	109.5
C10—C5—C6	120.54 (16)	H11A—C11—H11B	109.5
C10—C5—O1	119.79 (16)	O2—C11—H11C	109.5
C6—C5—O1	119.42 (16)	H11A—C11—H11C	109.5
C5—C6—C7	119.64 (16)	H11B—C11—H11C	109.5
			
C4—N1—C1—O1	179.38 (14)	C10—C5—C6—C7	−0.2 (3)
C4—N1—C1—C2	−0.8 (2)	O1—C5—C6—C7	−174.50 (14)
C5—O1—C1—N1	−2.0 (2)	C5—C6—C7—C8	−0.2 (3)
C5—O1—C1—C2	178.13 (14)	C11—O2—C8—C9	3.5 (2)
C3—N2—C2—C1	−0.2 (2)	C11—O2—C8—C7	−176.93 (14)
N1—C1—C2—N2	0.8 (3)	C6—C7—C8—O2	−179.41 (15)
O1—C1—C2—N2	−179.37 (15)	C6—C7—C8—C9	0.2 (3)
C2—N2—C3—C4	−0.3 (3)	O2—C8—C9—C10	179.76 (15)
C1—N1—C4—C3	0.3 (2)	C7—C8—C9—C10	0.2 (2)
N2—C3—C4—N1	0.3 (3)	C6—C5—C10—C9	0.6 (3)
C1—O1—C5—C10	90.59 (19)	O1—C5—C10—C9	174.88 (14)
C1—O1—C5—C6	−95.10 (18)	C8—C9—C10—C5	−0.6 (3)

### Hydrogen-bond geometry (Å, °)

**Table:**

Cg1 is the centroid of the N1,N2,C1–C4 ring.

**Table:**

*D*—H···*A*	*D*—H	H···*A*	*D*···*A*	*D*—H···*A*
C9—H9···Cg1^i^	0.93	2.87	3.6326 (18)	140

Symmetry codes: (i) −*x*+1, −*y*+1, −*z*+1.
